# Intelligence

**Published:** 1833-09

**Authors:** 


### INTELLIGENCE.

Prize Essays *proposed by the Medical Reform Association. For the Three best Essays on the following Subject, three Prizes are offered.*

*Subject.* "On the present State of the Medical Science and Practice* in the United Kingdom, and the most advisable and efficient mode of promoting the advancement and improvement of both in all these branches."

For the *best* essay will be awarded the sum of 50*l.* sterling.

For the *second,* the sum of 30*l.*

For the *third,* the sum of 20*l.*

*Conditions.* 1. The competition is open to *all* persons, whether of the medical profession or not, and the award will be made in *public.*

2. The essays are to be written in the English, French, or Latin languages, and these only.

3. They must be transmitted to Dr. Epps, 89, Great Russell street, Bloomsbury, London, on or before the 1st day of March, 1834.

4. They must be clearly and neatly written, and *not* in the handwriting of the authors.

5. Each essay is to bear a motto, and to be accompanied by a sealed letter, with a corresponding motto to that inscribed upon the essay. Within the sealed letter there must be the name and place of residence of the author.

6. None of the letters will be opened but those connected with the mottos of the successful essays; and the unsuccessful essays will be delivered, upon satisfactory reference, by Dr. Epps, the honorary secretary. The prize essays will be returned to their accredited authors, who may, if they think proper, publish them for their own advantage; or otherwise they will be published by the Association.

Signed, by order of the Association,

John Epps, m.d., *Honorary Secretary.*

n.b. One hundred pounds, the amount of the three prizes, are already lodged with the treasurer, Joseph Hume, Esq. m.p., who, with the other judges, will publicly deliver the several sums, as they shall be awarded to the successful candidates. The names of the other adjudicators will be published at a future and not distant period.—J. E.

* These comprise Medical Statistics.

### METEOROLOGICAL REGISTER,

By Messrs. HARRIS and Co., Mathematical Instrument Makers, 50, High Holborn.

| | Moon. | Rain | Thermometer. | | | Barometer. | | De Luc's Hygrometer. | | Winds. | | Atmospheric Variation. | | |
|---|---|---|---|---|---|---|---|---|---|---|---|---|---|---|
| July | | gauge. | 9 a.m. | max. | min. | 9 a.m. | 10 p.m. | 9 a.m. | 10 p.m. | 9 a.m. | 10 p.m. | 9 a.m. | 2 p.m. | 10 p.m. |
| 20 | | | 68 | 71 | 53 | 29.41 | 29.43 | 61 | 60 | NW | NW | fine | cloudy | cloudy |
| 21 | | | 62 | 66 | 54 | .49 | .52 | 60 | 63 | NW | W | cloudy | | |
| 22 | | | 60 | 67 | 58 | .52 | .47 | 65 | 65 | WSW | W | rain | fine | fine |
| 23 | ☽ | .14 | 62 | 68 | 52 | .50 | .64 | 67 | 66 | W | NW | showery | cloudy | fine |
| 24 | | .05 | 65 | 67 | 55 | .80 | .93 | 65 | 64 | NW | NW | | fine | fine |
| 25 | | | 65 | 70 | 55 | 30.03 | 30.03 | 64 | 62 | NW | NW | | | |
| 26 | | | 65 | 68 | 64 | .05 | 29.99 | 62 | 64 | NW | WNW | | | |
| 27 | | | 71 | 79 | 56 | .02 | 30.02 | 64 | 64 | WNW | N | | | |
| 28 | | | 62 | 77 | 60 | .02 | .02 | 64 | 64 | ESE | SE | | | |
| 29 | | | 69 | 79 | 57 | .05 | .11 | 64 | 62 | SE | ESE | | | |
| 30 | | | 57 | 70 | 54 | .15 | .21 | 62 | 63 | ENE | NE | cloudy | | |
| 31 | ○ | | 60 | 69 | 57 | .13 | .08 | 64 | 63 | NE | NE | fine | | |
| Aug. | | | | | | | | | | | | | | |
| 1 | | | 61 | 66 | 55 | .12 | .12 | 62 | 64 | ENE | NE | | | |
| 2 | | | 60 | 64 | 55 | .10 | .06 | 64 | 63 | NE | ENE | cloudy | | cloudy |
| 3 | | | 62 | 66 | 50 | .04 | .18 | 63 | 60 | NE | NE | | | fine |
| 4 | | | 59 | 65 | 57 | .02 | 29.93 | 60 | 61 | NE | NNE | fine | | |
| 5 | | | 63 | 66 | 58 | 29.84 | .83 | 59 | 59 | NW | ENE | | | rain |
| 6 | | .09 | 60 | 65 | 49 | .91 | .91 | 59 | 58 | ENE | ENE | | | fine |
| 7 | | | 60 | 66 | 55 | .86 | .81 | 58 | 58 | NE | ESE | | | |
| 8 | ☾ | | 63 | 70 | 60 | .81 | .83 | 58 | 59 | SE | ESE | | | |
| 9 | | | 67 | 73 | 58 | .77 | .76 | 59 | 58 | W | WNW | | | |
| 10 | | | 65 | 69 | 60 | .76 | .71 | 58 | 58 | NW | NW | | cloudy | |
| 11 | | | 66 | 70 | 51 | .81 | .85 | 59 | 60 | NNW | | | fine | |
| 12 | | | 61 | 66 | 55 | .92 | .81 | 60 | 58 | E | | | | cloudy |
| 13 | | | 60 | 63 | 56 | .56 | .44 | 58 | 61 | WNW | NNW | cloudy | | |
| 14 | | | 63 | 65 | 52 | .40 | .47 | 63 | 62 | ENE | NNE | fine | | fine |
| 15 | ● | | 60 | 66 | 55 | 50 | .52 | 60 | 59 | NE | NW | cloudy | cloudy | |
| 16 | | | 63 | 65 | 54 | .56 | .64 | 59 | 60 | NW | NE | fine | | cloudy |
| 17 | | | 61 | 66 | 58 | .65 | .59 | 60 | 57 | W | N | | fine | fine |
| 18 | | | 64 | 67 | 55 | .47 | .53 | 61 | 67 | W | NW | cloudy | cloudy | |
| 19 | | .12 | 63 | 68 | 58 | .57 | .64 | 65 | 63 | NW | NW | cloudy | fine | |

The quantity of Rain fallen in July, was 1 inch and 84-100ths.

